# A pipeline for processing hyperspectral images, with a case of melanin-containing barley grains as an example

**DOI:** 10.18699/vjgb-24-50

**Published:** 2024-07

**Authors:** I.D. Busov, M.A. Genaev, E.G. Komyshev, V.S. Koval, T.E. Zykova, A.Y. Glagoleva, D.A. Afonnikov

**Affiliations:** Institute of Cytology and Genetics of the Siberian Branch of the Russian Academy of Sciences, Novosibirsk, Russia Novosibirsk State University, Novosibirsk, Russia; Institute of Cytology and Genetics of the Siberian Branch of the Russian Academy of Sciences, Novosibirsk, Russia Novosibirsk State University, Novosibirsk, Russia; Institute of Cytology and Genetics of the Siberian Branch of the Russian Academy of Sciences, Novosibirsk, Russia; Institute of Cytology and Genetics of the Siberian Branch of the Russian Academy of Sciences, Novosibirsk, Russia; Institute of Cytology and Genetics of the Siberian Branch of the Russian Academy of Sciences, Novosibirsk, Russia Novosibirsk State University, Novosibirsk, Russia; Institute of Cytology and Genetics of the Siberian Branch of the Russian Academy of Sciences, Novosibirsk, Russia; Institute of Cytology and Genetics of the Siberian Branch of the Russian Academy of Sciences, Novosibirsk, Russia Novosibirsk State University, Novosibirsk, Russia

**Keywords:** hyperspectral images, machine learning, statistical analysis, barley grains, pigment composition, гиперспектральные изображения, машинное обучение, статистический анализ, зерна ячменя, пигментный состав

## Abstract

Analysis of hyperspectral images is of great interest in plant studies. Nowadays, this analysis is used more and more widely, so the development of hyperspectral image processing methods is an urgent task. This paper presents a hyperspectral image processing pipeline that includes: preprocessing, basic statistical analysis, visualization of a multichannel hyperspectral image, and solving classification and clustering problems using machine learning methods. The current version of the package implements the following methods: construction of a confidence interval of an arbitrary level for the difference of sample averages; verification of the similarity of intensity distributions of spectral lines for two sets of hyperspectral images on the basis of the Mann–Whitney U-criterion and Pearson’s criterion of agreement; visualization in two-dimensional space using dimensionality reduction methods PCA, ISOMAP and UMAP; classification using linear or ridge regression, random forest and catboost; clustering of samples using the EM-algorithm. The software pipeline is implemented in Python using the Pandas, NumPy, OpenCV, SciPy, Sklearn, Umap, CatBoost and Plotly libraries. The source code is available at: https://github.com/igor2704/Hyperspectral_images. The pipeline was applied to identify melanin pigment in the shell of barley grains based on hyperspectral data. Visualization based on PCA, UMAP and ISOMAP methods, as well as the use of clustering algorithms, showed that a linear separation of grain samples with and without pigmentation could be performed with high accuracy based on hyperspectral data. The analysis revealed statistically significant differences in the distribution of median intensities for samples of images of grains with and without pigmentation. Thus, it was demonstrated that hyperspectral images can be used to determine the presence or absence of melanin in barley grains with great accuracy. The flexible and convenient tool created in this work will significantly increase the efficiency of hyperspectral image analysis.

## Abstract (ru)

Анализ гиперспектральных изображений представляет большой интерес при изучении растений. В настоящее время такой анализ используется все более широко, поэтому создание методов обработки гиперспектральных изображений является актуальной задачей. В статье представлен конвейер для работы с гиперспектральными изображениями, который включает: предварительную обработку, базовый статистический анализ, визуализацию многоканального гиперспектрального изображения, а также решение задач классификации и кластеризации с применением методов машинного обучения. В текущей версии пакета программ реализованы следующие методы: построение доверительного интервала произвольного уровня для разницы выборочных средних; проверка сходства распределений интенсивности линий спектра для двух наборов гиперспектральных изображений на основе U-критерия Манна–Уитни и критерия согласия Пирсона; визуализация в двухмерном пространстве с применением методов понижения размерности PCA, ISOMAP и UMAP; классификация с использованием линейной или гребневой регрессии, случайного леса и градиентного бустинга; кластеризация образцов с помощью EM-алгоритма. Программный конвейер реализован на языке Python с использованием библиотек Pandas, NumPy, OpenCV, SciPy, Sklearn, Umap, CatBoost и Plotly. Исходный код доступен по адресу: https://github.com/igor2704/Hyperspectral_images. Данный конвейер был применен для идентификации пигмента меланина в оболочке зерен ячменя на базе гиперспектральных данных. Визуализация на основе методов PCA, UMAP и ISOMAP, а также использование алгоритмов кластеризации показали, что на базе гиперспектральных данных с высокой точностью можно провести линейное разделение образцов зерен с пигментацией и без нее. Анализ выявил статистически значимые различия в распределении медиан интенсивности для выборок изображений зерен с пигментом и без него. Таким образом, продемонстрировано, что с помощью гиперспектральных изображений с большой точностью можно определить наличие или отсутствие меланина в зернах ячменя. Созданный в данной работе гибкий и удобный инструмент позволит существенно повысить эффективность анализа гиперспектральных изображений.

## Introduction

The presence of pigments in the grain shell affects its various technological properties. For example, flavonoids, anthocyanins and carotenoids have a number of valuable properties, are antioxidants and affect the nutritional value of the grain. The addition of wheat bran with purple pericarp or blue aleurone layer to flour can improve the quality of bakery products through taste, texture and color characteristics (Machálková et al., 2017). Phlobaphenes, which impart red coloration to the grain pericarp, have a positive effect on the duration of grain dormancy and prevent preharvest germination (Flintham et al., 2002). Therefore, wheat genotypes with red grain coloration are used in breeding as donors of genes for resistance to preharvest grain germination (Krupnov et al., 2013; Fakthongphan et al., 2016).

Genetic control of color formation of both grains and other plant organs is carried out by genes encoding enzymes involved in pigment biosynthesis, as well as regulatory genes (Khlestkina, 2014; Lachman et al., 2017; Shoeva et al., 2018). For a number of pigments, these genes have been investigated quite well, to the point of fully deciphering their nucleotide sequences and location in the genome. However, for some pig-ments, such as melanin, which determines the black color-ation of barley grains, the molecular mechanisms of biosynthesis are not yet fully known (Glagoleva et al., 2017; Shoeva et al., 2018).

High-performance, non-destructive and accurate measurement techniques play an important role in assessing seed quality and improving agricultural production (Afonnikov et al., 2016, 2022). Hyperspectral and multispectral imaging techniques covering visible, near-infrared wavelength ranges provide spectral and spatial information for each image pixel. Hyperspectral images represent reflected intensity values for hundreds of wavelength intervals, which is significantly larger than for multispectral images with multiple wavelength ranges (Gowen et al., 2007).

By reducing the total amount of data, multispectral imaging systems aim to rapidly acquire images with relatively low spatial resolution and can be used in real time. Hyperspectral images, on the other hand, are typically used as datasets from which optimal wavelength ranges can be determined, which will be further used in multispectral imaging for a specific application problem (Qin et al., 2013). Such technologies allow obtaining more accurate information about the characteristics of reflected radiation of objects, compared to digital RGB images.

Hyperspectral data analysis has been successfully applied to crop yield estimation and prediction. L. Serrano et al. predicted biomass and yield of winter wheat using spectral indices (Serrano et al., 2000). W.S. Weber et al. (Weber et al., 2012) predicted grain yield using spectra (495–1,853 nm) of canopy and leaf reflectance of maize plants grown under different water regimes and obtained the most appropriate wavelengths for yield prediction. X. Zhang and Y. He (Zhang, He, 2013) developed a method for early and rapid seed yield estimation using hyperspectral images of oilseed rape leaves in the visible and near-infrared regions (380–1,030 nm). Soybean (Glycine max) seed yield was predicted based on hyperspectral data (395–1,005 nm) and machine learning algorithms: multilayer perseptron, support vector method and random forest, which also identified the most significant reflectance spectrum (395 nm) (Yoosefzadeh-Najafabadi et al., 2021).

Hyperspectral reflectance analysis can provide reliable information on seed viability of both weedy (Matzrafi et al., 2017) and cultivated plants: rice (He et al., 2019; Jin et al., 2022), wheat (Zhang et al., 2018), maize (Ambrose et al., 2016; Wakholi et al., 2018), peanut (Zou et al., 2023), melon (Kandpal et al., 2016), Japanese spinach mustard (Ma et al., 2020).

Based on hyperspectral technologies, innovative methods for diagnosing plant diseases are being developed (Cheshkova, 2022). Hyperspectral imaging technology covering the visible and near-infrared wavelength range (400–1,000 nm) was used to analyze rice to detect discolored, diseased seeds infected with bacterial panicle blight (Burkholderia glumae). It has been shown that determining the intensity of reflected radiation in a small number of wavelength bands is sufficient for accurate (> 90 %) classification of pathogen-affected and healthy plants (Baek et al., 2019).

Hyperspectral images are used to determine the chemical composition of seeds of cultivated plants. Near-infrared (895–2,504 nm) reflectance analysis has been shown to have potential in predicting anthocyanin content in black rice grains (Amanah et al., 2021). C. Liu et al. (Liu et al., 2020) demonstrated the feasibility of using near-infrared (930–2,500 nm) hyperspectral data analysis to determine the starch content of maize grains. G. Yang et al. (Yang et al., 2018) applied Raman hyperspectral technology with line scanning to determine the chemical composition of maize seeds. It was found that the characteristic Raman peaks identified at 477, 1,443, 1,522, 1,596 and 1,654 nm in the spectrum from 380 to 1,800 nm were associated with corn starch, oil and starch mixture, zeaxanthin, lignin and oil in corn seeds, respectively.

A method for non-destructive estimation of the concentrations and spatial distribution of moisture, protein and sugars at different developmental stages of vigna seeds has been proposed based on multispectral data from 20 discrete wavelengths in the ultraviolet, visible and near-infrared regions (ElMasry et al., 2022). Handheld near-infrared spectroscopy and hyperspectral imaging techniques have been used to quantify oil and fatty acid content and to classify seed species of the genus Brassica (da Silva Medeiros et al., 2022). Hyperspectral images have been used to solve the classification problem for grains of rice (Díaz-Martínez et al., 2023), ryegrass (Reddy et al., 2023) and many other crops important for the agricultural industry.

Platforms are being developed to provide hyperspectral information on seeds, such as HyperSeed, which includes a high-throughput line-scan spectrograph (600–1,700 nm) and open-source software based on a graphical user interface. The system was used to classify rice seeds (with 97.5 % accuracy) grown under heat stress and in control environments using both traditional machine learning and neural network (3D CNN) models (Gao et al., 2021).

Thus, the analysis of hyperspectral images is of great interest in various tasks related to plant research. However, developing algorithms to analyze such data is a time-consuming task.

This paper presents a hyperspectral image analysis pipeline, the use of which can significantly reduce the time cost in hyperspectral imaging-related research. We applied the developed pipeline to determine the melanin content of barley grains. Although the presence of melanin accounts for the dark coloration of the grain, in practice, visual determination of its presence is difficult. The dark color of the grain may be associated with the accumulation of anthocyanin pigments, which accumulate in the aleurone of the grain, giving ripe grains a gray color. Barley grains can also darken during storage. Therefore, accurate determination of the presence of melanin requires additional analysis, for example, immersion of grains in alkali solution for its extraction.

In this paper, we present a tool for hyperspectral image research, a pipeline, the use of which can significantly reduce time costs in such research. The capabilities of the developed pipeline are demonstrated on the example of the task of melanin content determination in barley grains. The task of studying the spectrum of melanin-containing and non-melanin-containing grains was chosen for testing, since it is known that there are significant differences in their spectrum. Our analysis also showed significant differences in the spectrum of grains containing melanin and samples without this pigment. Unlike other works in this area, in addition to classifying the samples, we had the task of implementing a pipeline to facilitate and automate the acquisition of hyperspectral images. The developed pipeline allows us to visualize and cluster the input data, as well as to perform their statistical analysis.

## Materials and methods

Plant material. Seeds of 313 barley (Hordeum vulgare) accessions were selected for the study, of which 117 accessions contained melanin and the remaining 196 accessions lacked this pigment (Supplementary Material)1. The material was obtained from the barley collection of the All-Russian Institute of Plant Genetic Resources named after N.I. Vavilov (VIR, https://www.vir.nw.ru), barley collection of the Institute of Cytology and Genetics of the Siberian Branch of the Russian Academy of Sciences (ICG, http://www.bionet.nsc.ru). Material from the Oregon Wolfe Barleys population (OWB, https://barleyworld.org/owb) was also used. Biochemical ana-lysis of samples with stained grain, as well as a detailed description of the melanin detection method were performed by A.Y. Glagoleva et al. (Glagoleva, et al. 2022).


Supplementary Materials are available in the online version of the paper: https://vavilov.elpub.ru/jour/manager/files/Suppl_Busov_Engl_28_4.pdf


Chemical method for determination of pigment composition of grains. To determine the qualitative presence of melanin in the grain, extraction with 2 % NaOH followed by blackening of the solution was performed. Based on this method, each of the samples was assigned a pigmentation type based on the presence of pigment: “contain melanins” or “do not contain melanins”.

**Fig. 1.**
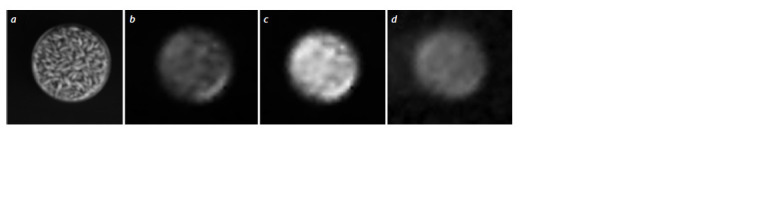
Image of barley grains in a Petri dish in shades of gray (a) and visualization of reflected radiation intensity in the wavelength intervals of 450 nm (b), 554 nm (c), and 986 nm (d).

Image acquisition. Hyperspectral images of grains were obtained using a Cubert S185 camera with a Cinegon 1.8/16 lens. For this purpose, a plastic petri dish with a diameter of 55 mm filled with grains without gaps was placed on a white matte sheet of A3 paper. A diffusing light was placed on the sides, and the camera was fixed on a tripod from above, with the lens vertically downward. At the output, the camera produced a 138-channel hyperspectral image, each channel of which corresponded to the reflection intensity in a certain wavelength range (Fig. 1). The size of the hyperspectral image was: 50 by 50 pixels, spectral range: 450–998 n.m., spectral channel width: 4 n.m. The images were saved in tiff format.

Thus, the hyperspectral image obtained by a Cubert S185 camera is a hypercube, in which indices i, j (i, j = 1, ... 50) correspond to spatial coordinates (image pixels), index k = 1, ... 138, corresponds to hyperspectral lines with a certain wavelength. Each element of this hypercube corresponds to the intensity of reflected radiation from the subject for a pixel in the image with spatial coordinates i, j and spectral line with serial number k.

Images for the study of the pigment composition of barley grains were obtained from several series of surveys over several days.

Pipeline description. The input data for the pipeline are hyperspectral images in tiff format described in the previous section and calibration hyperspectral images (black and white background images in tiff format).

**Fig. 2.**
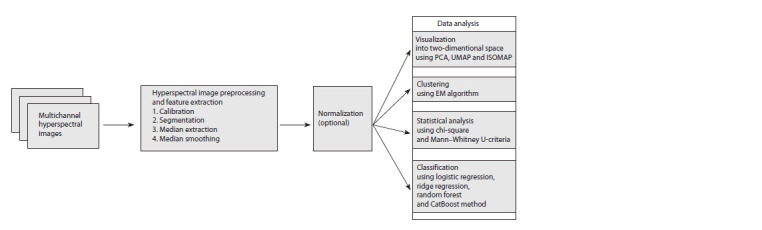
Pipeline schematic for hyperspectral image analysis.

Multichannel hyperspectral image analysis is performed in several steps including preprocessing, feature extraction, normalization and direct data analysis (Fig. 2).

Hyperspectral image preprocessing and feature extraction. The nature of ambient light can affect the reflected spectrum intensities (Zahavi et al., 2019). In order that the reflected emission intensities on different spectrum lines could be compared for different imaging conditions, we used image calibration according to the following formula where Sijk is the barley hyperspectral image hypercube element, Dijk is the black background calibration image element, Wijk is the white background calibration image element, Rijk is the calibrated image element.

**Formula. 1.**
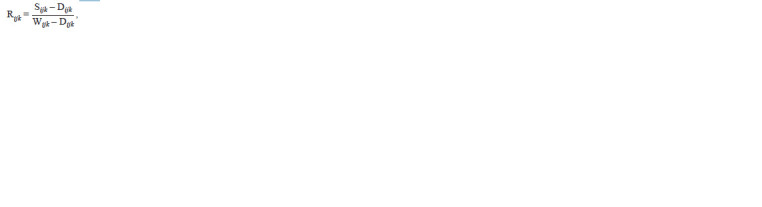
Formula. 1.

The calibrated images are converted to a three-channel image approximating RGB based on intensities for wavelengths 450 nm (blue), 510 nm (green), 630 nm (red) using a threshold transformation (OpenCV library threshold() function (Howse J., 2013)). This image is converted to a grayscale image (OpenCV library function cvtColor()) and binarized to highlight the Petri dish region with grains. If necessary, the pipeline allows you to use your own implementation of segmentation, but for the task at hand, segmentation by threshold value is sufficient.

Then, for each image, the medians for each hyperspectral channel are calculated from the pixel values in the segmented area occupied by grains. The Savitzky–Golay filter (Savitzky, Golay, 1964) is used to smooth the median values. The obtained vector of medians characterizes hyperspectral data for each studied sample.

Normalization. In order to eliminate differences arising between imaging series, 2 methods of image normalization were implemented in the pipeline. The first way of normalization is standardization (subtraction of the sample mean and division by standard deviation) by identical samples of each image (vectors of medians). The second method is standardization by identical groups (samples containing/not containing melanin), within each series.

Data analysis. Dimensionality reduction methods. The pipeline uses 3 dimensionality reduction methods: PCA (principal component analysis) (Jolliffe, 2002), ISOMAP (isometric mapping) (Balasubramanian, Schwartz, 2002), and UMAP (uniform manifold approximation and projection) (McInnes, et al., 2018) to visualize samples clearly in hyperspectral data space. PCA is a linear dimensionality reduction method that preserves the largest percentage of variance.

ISOMAP, UMAP are nonlinear dimensionality reduction methods. The UMAP method builds a weighted graph where only the nearest neighbors are connected by edges (the number of neighbors is given as a pipeline parameter). The ISOMAP method first constructs a sparse graph where, just as in the graph for UMAP, only the nearest neighbors are connected by edges (the number of neighbors is given as a pipeline parameter). Then, either the Dijkstra algorithm (Cormen et al., 2002) or the Floyd–Worshall algorithm (Cormen et al., 2002) is used to compute the distances between objects in the sparse graph for the ISOMAP method. After constructing the graphs and the distance matrix for them, the UMAP and ISOMAP methods are used to determine the position of the samples in a space of lower dimensionality (usually 2 or 3) that preserves the distances between objects. The dimensionality reduction methods were implemented using the Sklearn (Hao et al., 2019) and Umap (Becht et al., 2019) libraries.

Visualization. After the preprocessing and feature extraction stages, each sample (hyperspectral image) is presented as a vector lying in a dimensionality space equal to the number of hyperspectral image channels. The elements of the vector correspond to the reflected radiation intensity for the corresponding channel. After obtaining the coordinates of the samples in lower dimensionality spaces, visualization in the form of a scatter diagram was performed using the plotly.express. scatter function of the Plotly library (Stančin I. et al., 2019).

Clustering. The pipeline implemented clustering using the EM algorithm (Dempster et al., 1977). It was assumed that each sample could belong to each cluster with a probability obeying the Gaussian distribution mixture model. The parameters of the distributions were found using the maximum likelihood method, using the EM algorithm. The main hyperparameters of clustering are: dimensionality of the space in which clustering takes place, method of dimensionality reduction, method of initialization of weights (random initialization, initialization by the k-means method). The pipeline returns a table with information about the most frequent group in each cluster and the percentage of samples in it. The Sklearn library was used to implement clustering.

Statistical analysis. In the created pipeline for the difference of sample averages of two groups of images, it is possible to determine the confidence interval at a given level of significance, which is based on the central limit theorem (CLT). According to the CLT, if the sample size is sufficient, we can assume that the difference of sample averages is normally distributed. For this random variable, the sample mean and sample variance are calculated, and thus confidence intervals of arbitrary level are constructed.

Tests based on the Mann–Whitney U-criterion (Wilcoxon, 1945) and chi-square criterion (Greenwood, Nikulin, 1996) were added to the pipeline to test the hypothesis that the distributions of the two groups coincide. Statistical analysis was implemented using the SciPy library (Nunez-Iglesias et al., 2017).

Classification. The developed pipeline classifies hyperspectral images using methods such as logistic regression (Norman, Harry, 2007), ridge regression (Norman, Harry, 2007), random forest (Ho,1995) and gradient boosting (Prokhorenkova et al., 2017). The pipeline returns tables with classification results on metrics such as accuracy, F1, precision and recall, as well as error matrices for each classifier. The first table contains classification results for macro metrics and the second, for micro metrics. If a function that converts a group into a vector is passed to the pipeline, the pipeline returns a third table with the averaged binary classification results for each individual component of the vector. Classification is implemented using the Sklearn and CatBoost libraries (Hancock, Khoshgoftaar, 2020).

## Results

Sample images for pigment composition analysis were obtained from three series of surveys. In two series, grains containing melanin were absent. In one series, both grains with melanin and grains without this pigment were present. There were no identical samples in different series of imaging. For each sample, two images were obtained: a hyperspectral image and a high-resolution image. Since samples without pigment were present in all imaging series, normalization by samples of grains with no pigment was performed.

Median graph

**Fig. 3.**
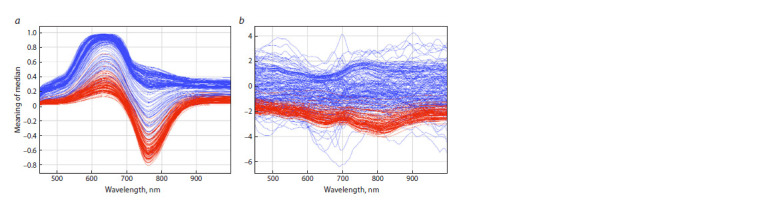
Graph of the dependence of the median intensity of reflected radiation for barley grain samples as a function of wavelength. On the left is the graph (a) without normalization, on the right is the graph (b) of medians after normalization by identical groups. Blue lines correspond to medians of images of barley grains without melanin, and red lines, to medians of images of grains with melanin

The obtained medians were used to plot the dependence of intensity on wavelength for each image (Fig. 3). As can be noted, the hyperspectrum of grains containing melanin differs markedly from the hyperspectrum of grains without this pigment.

The plot without normalization for the median curves shows local maxima in the 600–700 nm range, and local minima in the 700–800 nm range. Most of the median curves of grains with melanin are more tightly clustered (wavelength-averaged dispersion is smaller) and have smaller mean values than the curves of samples without pigment over the entire wavelength range. Despite the partial overlap, most of the median curves of the samples with pigment are distinguishable from the median curves of the samples without pigment

Visualization in two-dimensional space

**Fig. 4.**
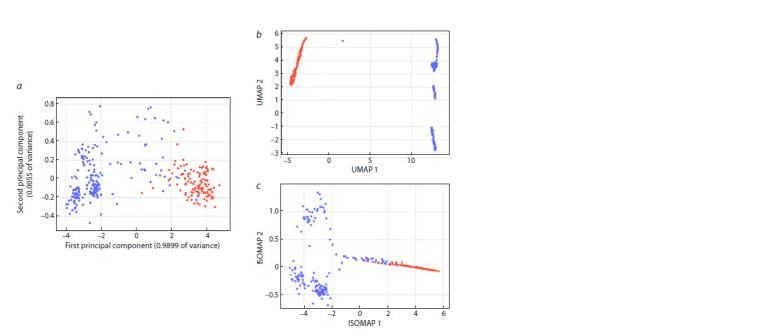
Plots of distributions in two-dimensional space obtained with PCA (a), UMAP (b) and ISOMAP (c). Blue points correspond to samples without melanin and red points, to samples with melanin.

In the PCA (Fig. 4a) and ISOMAP (Fig. 4c) plots, it can be observed that the dispersion in grains without melanin is larger than in grains with this pigment

Clustering results

**Fig. 5.**
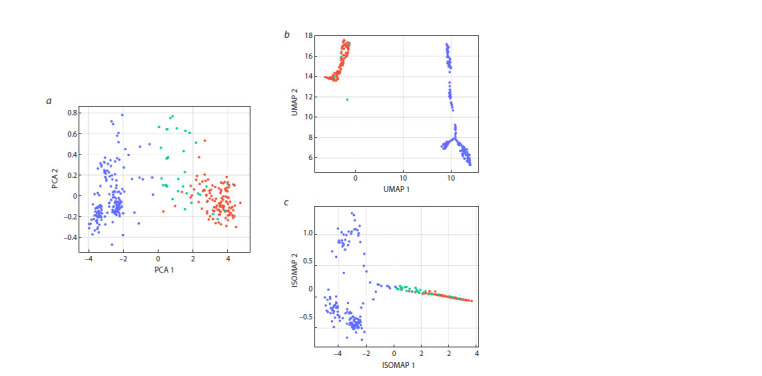
Visualization of the clustering results using the EM algorithm. Initialization was performed using the k-means method in a space of dimensionality 15, using the dimensionality reduction methods PCA (a), UMAP (b) and ISOMAP (c). Blue dots correspond to grains that do not contain melanin and are in the first cluster. Red dots correspond to grains that contain melanin and are in the second cluster. Green dots stand for grains that do not contain melanin and belong to the second cluster. Samples containing melanin but assigned to the first cluster were absent.

Clustering was performed into 2 clusters representing samples with melanin and samples without pigment. Clustering confirms that the medians of hyperspectral images are separable with high accuracy (Fig. 5, Table 1).

**Table 1.**
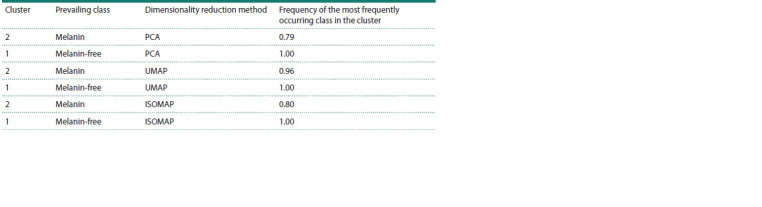
Clustering accuracy by the EM algorithm with random initialization in dimension space 15, using the UMAP dimensionality reduction method Notе. The prevalent class is the most frequent class of samples in the cluster

The samples with and without pigment were least clearly separated in the PCA plot (Fig. 4a): samples without pigment (blue dots) are present near the cluster of samples with melanin (red dots on the right). These samples were assigned to the second cluster (green dots) during clustering (Fig. 5a). In contrast, in the UMAP plot, all samples with pigment were arranged in isolation (Fig. 4b), on the top left, while samples without pigment formed clusters of dots on the right. However, in the clustering plot (Fig. 5b), single samples on the left were assigned to the second cluster. The ISOMAP plot (Fig. 4c) shows a good clustering of samples with melanin, while samples without pigment were distributed on the left, and to a lesser extent, on the right side of the plot, partially overlapping with samples with melanin. In clustering (Fig. 5c), some of these samples were assigned to the second cluster (green dots in the right part of the graph).

Table 1 numerically confirms that the median vectors of hyperspectral images of grains of different classes (containing and not containing melanin) in clustering mainly fall into different clusters, which indicates the existence of significant differences in the spectrum of grains with and without pigment. It is also worth noting that the first cluster includes samples exclusively without melanin.

Statistical analysis

**Fig. 6.**
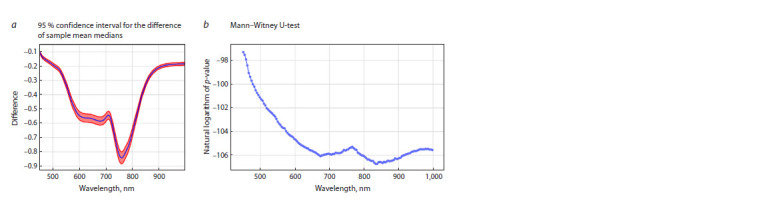
(a) 95 percent confidence interval for the difference of sample mean medians. The blue line is the values of the difference of sample mean differences. The red area is the 95 percent confidence interval. (b) Logarithm p-value plot for the Mann–Whitney U-criterion for the difference in mean values of reflected spectrum intensity for grain samples with and without melanin for different wavelength intervals.

Figure 6a shows the differences of sample mean values of reflected radiation intensity for barley samples for all wavelength intervals. As can be noted, the mean values of different groups of grains are statistically significantly different in the whole wavelength interval under consideration. Figure 6b shows a plot of the dependence of the logarithm of the reliability of differences ( p-value) on wavelength for the Mann–Whitney U-criterion. This criterion (taking into account the Bonferroni correction) allowed us to detect statistically significant differences for the entire hyperspectrum under study.

Classification results

The task of classifying hyperspectral grain images based on melanin content is a binary classification task. Table 2 shows the classification accuracy estimates for accuracy, F1, precision and recall metrics for each dimensionality reduction method. The test sample size was 47 samples and the training sample size was 266 samples. The k-fold cross validation (k = 4) was used in training. 18 samples in the test sample contained melanin; 29 samples were without melanin; 99 samples in the training sample were with melanin; 167 samples were without this pigment

**Table 2.**
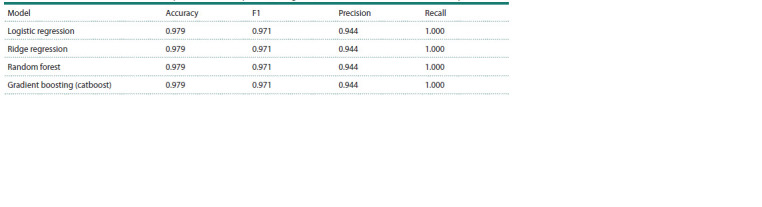
Classification results of the test sample in dimension space 15, using PCA, UMAP and ISOMAP for dimensionality reduction Notе. For the obtained training and test samples, the results when using different dimensionality reduction methods on the test sample were the same.

The studied grain samples contained anthocyanins in addition to melanin, which allowed us to study the possibility of differentiation between melanins and anthocyanins. Samples were classified in the 15-dimensional space previously obtained by PCA using logistic regression (266 samples for the training sample and 47 samples for the test sample). As a result, classification errors occurred mainly between the classes “without pigments” and “with anthocyanins only”, as well as in identifying samples containing both pigments and grains containing only melanin (Fig. 8).

**Fig. 7.**
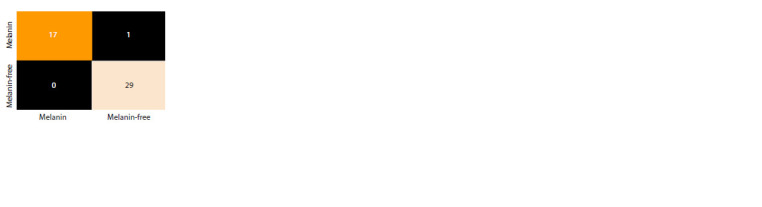
Error matrix for the test sample in dimension space 15. For the obtained training and test samples, the results using different dimensionality reduction methods and different classification models on the test sample were the same.

**Fig. 8.**
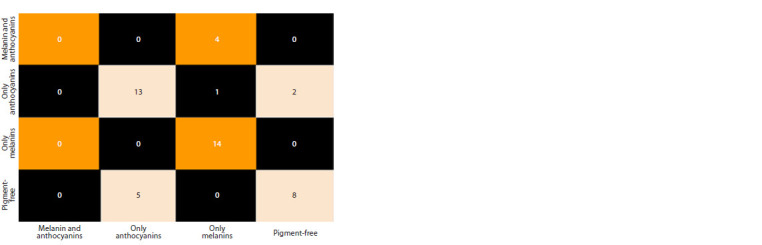
Classification error matrix based on logistic regression of grain samples into 4 classes: containing melanin and anthocyanins, only anthocyanins, only melanin and without pigments.

**Fig. 9.**
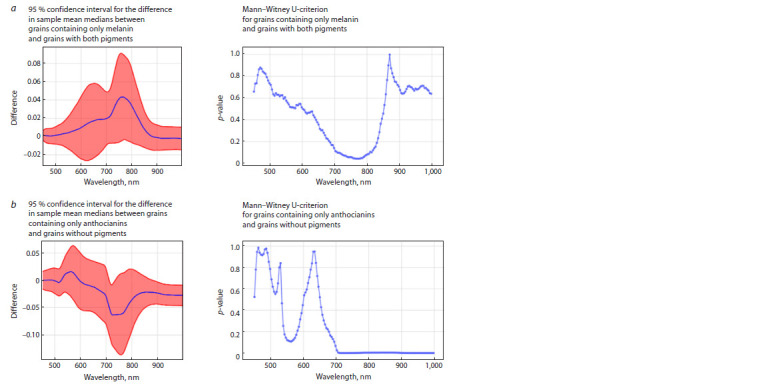
Plots of the logarithm of the p-value for the Mann–Whitney U-criterion for the difference in mean values of reflected spectrum intensity and 95 percent confidence intervals for the difference in sample mean medians.

Based on the results of the statistical analysis, no statistically significant differences ( p-value < 0.05/138, taking into account the Bonferroni correction) were found across the spectrum for grains containing only melanin and grains with both pigments. The lowest p-value for the Mann–Whitney criterion for these groups was reached at 774 nm and was 0.0438 (Fig. 9a). For grains containing only anthocyanins and grains without pigments, statistically significant differences ( p-value <0.05/138, taking into account the Bonferroni correction) were found at wavelengths falling in the red and infrared bands (> 714 nm) (Fig. 9b).

## Discussion

Pipelines in the field of hyperspectral image processing

There are many state-of-the-art approaches to automate the process of hyperspectral data analysis. They utilize a wide range of machine learning, computer vision and advanced data processing techniques. Hyperspectral images are characterized by high dimensionality, large data volume, are affected by noise, require calibration and normalization, and are more difficult to visualize compared to RGB images. In addition, there is a problem of training sample size. To solve this prob-lem, various methods of increasing the size of training sets (augmentation) are used. On the other hand, the high dimensionality of hyperspectral data can easily lead to a high level of data redundancy. To solve this problem, algorithms for ranking and filtering significant features, as well as for selecting groups of significant spectra are used.

The acquired hyperspectral raw data are preprocessed: outlier detection using principal component analysis (PCA), group averaging, scaling and centering (Yoosefzadeh-Najafabadi et al., 2021); calibration of the acquired images using reference images (dark and white); normalization; Savitzky– Golay filtering; and parameter ranking and filtering for classification to improve model accuracy and generality (Amanah et al., 2021).

The use of dimensionality reduction techniques may lead to a decrease in classification accuracy, however, it may be justified in order to increase the generality of the models – to avoid overfitting them. Thus, the development of approaches for solving individual problems using hyperspectral data requires multi-stage processing, the realization of which is possible in a software pipeline architecture, where each individual stage is replaceable and can be carefully tuned and adapted.

To solve such problems, pipeline approaches are currently being actively developed. For example, in the work of F. Zhu et al. (Zhu et al., 2024), the authors investigated ways to preprocess spectral data to effectively reduce the effect of different illumination on chlorophyll estimation in basil crops grown under different light intensities. The authors determined the optimal analysis pipeline for near-field hyperspectral imaging data by evaluating the performance of regression modeling and obtaining satisfactory chlorophyll distribution maps consistent with observed differences in chlorophyll levels

In their work, H. Feng et al. (Feng et al., 2017) developed an integrated image analysis pipeline for automatic processing of large volumes of hyperspectral data. Models were built to accurately quantify 4 pigments (chlorophyll a, chlorophyll b, total chlorophyll, and carotenoids) from rice leaves and identified important wavelength groups (700–760 nm) associated with these pigments. At the tillering stage, the R2 values and mean absolute percentage errors of the models were 0.827–0.928 and 6.94–12.84 %, respectively.

By establishing a four-stage image processing and data analysis management pipeline, the applicability of hyperspectral remote sensing for early detection of drought stress and root-knot nematodes (RKN) infestation in tomato plants was evaluated (Žibrat et al., 2019). The pipeline included: image acquisition, data extraction, preprocessing and analysis. By combining discriminant analysis based on partial least squares and support vector machine with time series analysis, the authors achieved 100 % classification success in determining irrigation regime and infestation rate. Thus, the development of pipelined solutions for hyperspectral data analysis is an actively developing area at the moment.

The hyperspectral data analysis example presented in this paper also uses a pipeline approach, which includes preprocessing and dimensionality reduction data analysis (principal component analysis, group averaging, calibration using reference images, normalization, Savitzky–Golay filtering). The pipeline structure allows the use of different dimensionality reduction methods: PCA (Jolliffe, 2002), ISOMAP (Balasubramanian, Schwartz, 2002) and UMAP (McInnes, et al., 2018) in combination with different classification methods: logistic regression (Norman, Harry, 2007), ridge regression (Norman, Harry, 2007), random forest (Ho, 1995), gradient boosting (Prokhorenkova et al., 2017).

Methods of plant image classification based on hyperspectral data

Hyperspectral images are used to classify the physiological state of plants. T. Zhang et al. (2018) investigated the feasibility of using hyperspectral imaging techniques in the visible and near-infrared ranges (VIS/NIR, 400–1,000 nm) to recognize viable and non-viable wheat seeds. For this purpose, classification models, partial least squares discriminant analysis (PLS-DA) and support vector machines (SVM) combined with some preprocessing techniques and sequential projection algorithm (SPA) were used. The results showed that the standard normal variation (SNV)-SPA-PLS-DA model had high classification accuracy for whole seeds (> 85.2 %) and viable seeds (> 89.5 %).

Y. Lu et al. (Lu et al., 2022) were able to achieve up to 99.6 % accuracy in differentiating five cannabis varieties, and 100 % accuracy in distinguishing between five growth stages and two plant organs (leaves and flowers) using a desktop hyperspectral imaging system in the spectral range of 400– 1,000 nm and machine learning based on regularized linear discriminant analysis.

The work published by B.C. da Silva et al. (2024) evaluated the performance of five ML algorithms and the sensitivity of 90 spectra in the task of predicting the content of nitrogen and pigments (chlorophyll and carotenoids) in maize leaves at different phenological stages to optimize nitrogen fertilization. In predicting the contents of chlorophyll a and b, the value of Pearson correlation coefficient between predicted and observed data was about 0.6, and the mean absolute error (MAE) was below 0.5. When flavonoid content was predicted, the value of the correlation coefficient between predicted and observed data was about 0.6 and the MAE was 0.07. When nitrogen content was predicted, the correlation coefficient values were above 0.35 and the MAE was below 2.75.

In the paper published by Changyeun Mo et al. (2014), the authors developed a method to assess the viability of pepper (Capsicum annuum L.) seeds based on hyperspectral imaging in the 400–700 nm range obtained using blue, green, red and RGB LED illumination. For this purpose, a partial least squares discriminant analysis (PLS-DA) model was developed based on the standard normal variant of RGB LED illumination (400–700 nm), which provided recognition accuracies ranging from 96.7 to 100 %.

R. Falcioni et al. (2023) developed a method to estimate pigments such as chlorophylls, carotenoids, anthocyanins and flavonoids in six agronomic crops: maize, sugarcane, coffee, rapeseed, wheat and tobacco based on hyperspectral data. Clustering based on principal component analysis (PCA) and Kappa coefficient analysis yielded accuracies ranging from 92 to 100 % in the ultraviolet (UV-VIS), near-infrared (NIR) and shortwave infrared (SWIR) bands.

In our study, we obtained quite high precision values: accuracy = 0.979, F1 = 0.971 with precision = 0.944 and recall = 1.000 for all prediction models, which is comparable to similar values in other works, in particular, those described above. The resulting estimates were similar for all models, probably due to the fact that the sample size was small and homogeneous. As a result, with the resulting partitioning, all models in the test sample made one error, misclassifying one sample. On the other hand, this demonstrates the high stability of the predictions based on hyperspectral data and the proposed models.

In our previous work (Komyshev et al., 2023), we developed a method for estimating the presence of anthocyanins and melanin in barley grain shells based on the analysis of digital RGB images using computer vision and machine learning algorithms. We used a similar imaging protocol using Petri dishes for grains, but imaging was performed with a conventional RGB camera. The samples were taken from a similar collection. In that case, the best accuracy (accuracy = 0.821) was shown by the U-Net model based on the EfficientNetB0 topology. Thus, even when using deep machine learning methods, the classification accuracy was lower than in the present work. It can be concluded that more hyperspectral images allow more accurate classification of plant grains by pigment content using less resource-intensive “shallow” machine learning methods.

We studied the effect of the presence of anthocyanins on the accuracy of melanin determination in barley samples. The accuracy of melanin determination in samples containing anthocyanins was lower (accuracy = 0.95) compared to samples without this pigment (accuracy = 1) (Fig. 8). Thus, the presence of anthocyanins insignificantly reduces the accuracy of melanin determination in samples

The ability to differentiate samples with only melanin from those with both melanin and anthocyanins was poor (Fig. 9a). Determination of anthocyanins, based on the hyperspectral data obtained, seems to be possible with high accuracy due to the spectrum in wavelengths falling in the red and infrared ranges (> 714 nm) (Fig. 9b). Thus, this approach allows differentiating grains without pigments from grains with anthocyanins, but does not allow determining the presence of anthocyanins in samples with melanin.

Our goal was to explore the possibility of distinguishing between melanin-containing and non-melanin-containing seed samples using hyperspectral data alone. We also tested several approaches consisting of interchangeable methods that form a typical hyperspectral data processing pipeline and formed it into a software tool. This software tool can be used to quickly build a hyperspectral data analysis algorithm that includes the main data processing steps such as image loading, preprocessing, analysis and visualization.

## Conclusion

Visualization based on the PCA, UMAP and ISOMAP methods, as well as clustering in dimension space 15, showed that barley samples with and without melanin could be divided into two respective classes with high accuracy on the basis of hyperspectral images. The analysis revealed statistically significant differences in the distribution of reflected intensity for these samples for all hyperspectral lines.

Advantages of using the developed pipeline over classical and more accurate biochemical methods of solving the classification problem are low time and labor costs, as well as objectivity of the obtained results. Neural networks/deep machine learning methods were not used in this version of the package for classification. The disadvantages of neural network approaches compared to the methods implemented in the pipeline may be the difficult interpretability of the prediction results, as well as the need for a training sample of a very large volume.

In this paper, an open-source Python-based computational pipeline has been developed for hyperspectral image analysis, which includes visualization in two-dimensional space, clustering, basic statistical analysis and classification. The proposed software package can significantly reduce the time cost in studies involving hyperspectral image analysis. The developed pipeline was tested in the task of investigating the effect of melanin on the hyperspectrum of barley grains.

## Conflict of interest

The authors declare no conflict of interest.
